# Abusive head trauma in children under 2 years of age in Latin America: a systematic review

**DOI:** 10.1007/s00381-026-07236-6

**Published:** 2026-03-31

**Authors:** José Roberto Tude Melo, Maria Antonia Coladeti Fernandes, Júlia Calviello Giordano, Isabela Zampirolli Leal, Caio Vinicius de Almeida Chaves, José Carlos Esteves Veiga

**Affiliations:** 1 Santa Casa of São Paulo School of Medical Sciences, São Paulo, Brazil; 2 Present Address: Division of Neurosurgery, Department of Surgery, Santa Casa of São Paulo School of Medical Sciences, São Paulo, Brazil

**Keywords:** Child, Traumatic brain injury, Child maltreatment, Domestic violence, Mortality

## Abstract

Among the types of violence against children, abusive head trauma (AHT) is recognized as one of the main causes of violent death and neurological disabilities in infants. The scope of this systematic review was to examine the scenario and knowledge about AHT in Latin American countries based on studies conducted in Latin America and/or published by Latin American researchers between 2010 and 2024 (15 years). This systematic review was conducted in the PubMed/NIH (National Library of Medicine; National Center for Biotechnology Information), SciELO (Scientific Electronic Library Online), and LILACS (Latin American and Caribbean Literature in Health Sciences) databases. This systematic review was recorded in the Prospective International Registry of Systematic Reviews (PROSPERO CRD42025628791). Among the 462 articles found in the databases, 23 studies were published by Latin American researchers. Following the Preferred Reporting Items for Systematic Reviews and Meta-Analyses (PRISMA) guidelines, only 7 articles met the eligibility criteria. Among children with traumatic brain injury, the presence of seizures (70% vs. 46%; *p* < 0.0001), retinal hemorrhages (78% vs. 3%; *p* < 0.0001), and subdural hematoma (91% vs. 29%; *p* < 0.0001) prevailed in suspected or confirmed cases of AHT. There is a shortage of scientific articles on AHT with sufficient sample size to allow us to know the geographic distribution of this event in Latin America. However, Latin American researchers recognize an unwitnessed or incoherent history of trauma along with a lowered level of consciousness, seizures, retinal hemorrhages, and subdural hematoma as strong indicators for suspicion of AHT.

## Background

Latin America comprises 20 low- and middle-income countries with approximately 570 million inhabitants. This geographic region encompasses Argentina, Bolivia, Brazil, Chile, Colombia, Costa Rica, Cuba, Ecuador, El Salvador, Guatemala, Haiti, Honduras, Mexico, Nicaragua, Panama, Paraguay, Peru, Dominican Republic, Uruguay, and Venezuela [1]. The European occupation and domination of Latin America was characterized by the use of violence, especially in the case of the Portuguese and Spanish colonization. Violence was at times trivialized and considered inseparable from the “civilizing process,” remaining ingrained in the structure of some societies until present [2, 3]. For many years, violence has been used by parents as a method of punishing children to “educate” them [4]. Regarding violence against children, non-accidental or inflicted abusive head trauma (AHT) stands out worldwide as the main cause of neurological disabilities and violent death in infants [5–7]. Given that most research on AHT has been carried out in rich and developed countries, the scope of this systematic review was to examine scientific articles published and indexed in the PubMed/NIH (National Library of Medicine; National Center for Biotechnology Information), SciELO (Scientific Electronic Library Online) and LILACS/BVS (Latin American and Caribbean Literature in Health Sciences—Complete collection from the Regional Portal of the Virtual Health Library) databases to identify aspects related to the epidemiological characteristics, management for diagnosis and treatment of AHT in Latin America and synthesize the information acquired in the case of multicentric studies or other forms of international cooperation.

## Methods

### Study design, sources of information, and ethical considerations

We analyzed scientific articles published between 2010 and 2024 (15 years) available in the PubMed/NIH, SciELO and LILACS/BVS databases. The search for articles was made using terms of interest in the theme investigated, including the analyzed population (children ≤ 2 years of age) and the trauma mechanism investigated (AHT). Only scientific articles published by Latin American researchers and/or carried out in the Latin American territory that were evaluated and authorized by research ethics committees were included in this systematic review. As this study was designed exclusively as a systematic review and the data are de-identified, this study was exempt from institutional review board approval or consent by a research ethics committee.

### Selection of studies (eligibility criteria)

Considering the different terms used to define AHT, as well as recognizing subdural hematoma (SDH) as the main intracranial lesion described in these victims [5–7], the following terms were used in the search: “abusive head trauma” or “abusive head injury” or “shaken baby” or “non-accidental head injury” or “non-accidental head trauma” or “non-accidental traumatic brain injury” or “non-accidental traumatic head injury” or “non-accidental traumatic head trauma” and “subdural hematoma.” The electronic search for articles followed the guidelines of the Preferred Reporting Items for Systematic Reviews and Meta-Analyses (PRISMA) guidelines [8], and the ROBIS tool [9] was used to assess the risk of bias. This systematic review was recorded in the Prospective International Registry of Systematic Reviews (PROSPERO CRD42025628791) [10].

In the first stage of the study (first-level screening), 5 authors (JRTM, MACF, JCG, IZL, CVAC) read the titles of all citations of articles found and removed the duplicates as well as articles that did not address the main theme or which were written in languages other than English, Spanish or Portuguese, considering the region of interest for the study. The second stage (second-level screening) consisted of the reading of the abstracts of the selected references in order to choose articles exclusively addressing traumatic brain injury (TBI) in the pediatric population and including in their samples children with suspected or confirmed AHT. We included articles addressing “suspected AHT” because, in some cases, the authors cannot mention the confirmation of the abuse because such a statement requires a legal decision after a trial in a sphere outside the competence of health professionals [11]. At this stage, we selected articles whose author or first co-author was Latin American. If all authors were from regions outside Latin America, then the article was selected only if it addressed the Latin American population. This information was checked by reading the abstract and the institutional affiliation of the authors. In the third stage (third-level screening), all authors read the selected articles in full length.

**Fig. 1:**
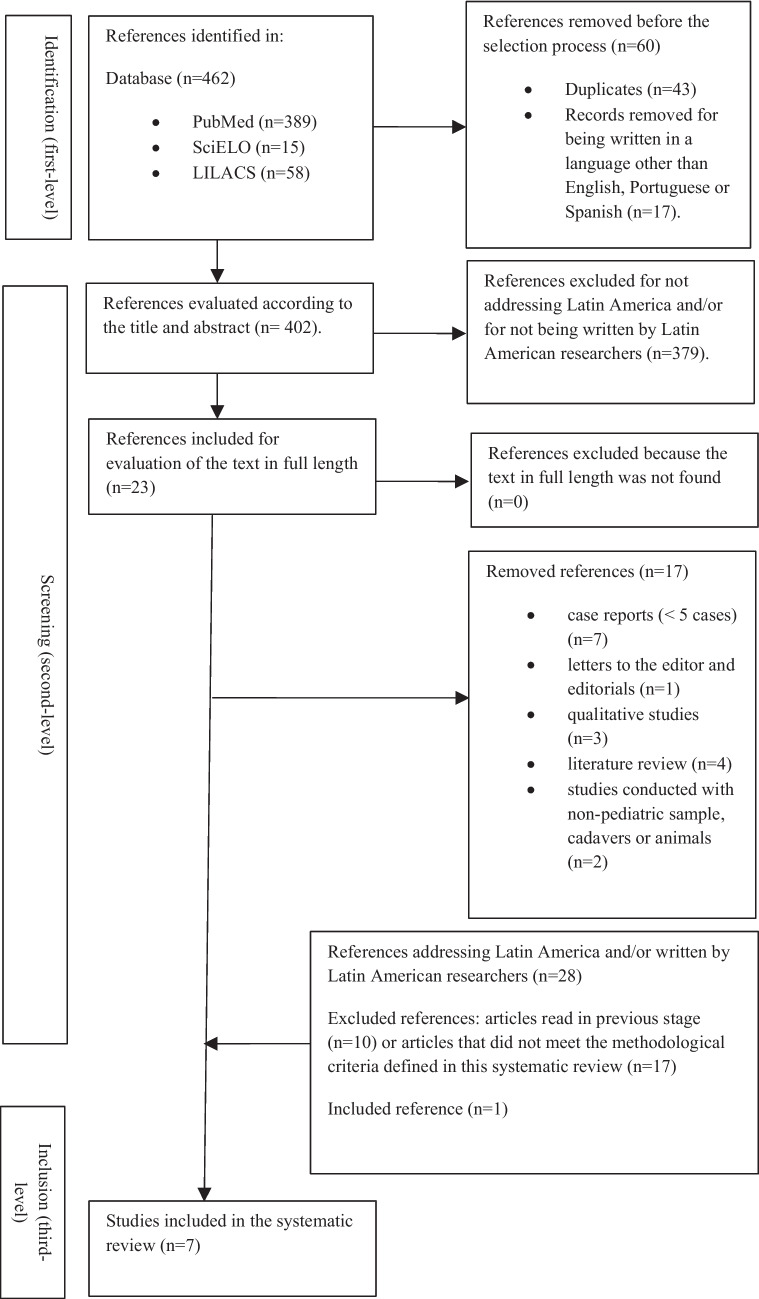
Flowchart following the PRISMA (Preferred Reporting Items for Systematic Reviews and Meta-Analyses) criteria for selection of bibliographic references to be included in the present systematic review on abusive head trauma (AHT) in children ≤ 2 years of age in Latin America published between 2010 and 2024

As for the type of articles, those classified as original research (retrospective or prospective cohort, matched-pair case control, cross-sectional studies) or case series (≥ 5 cases) published in peer-reviewed journals with samples including children ≤ 2 years of age with suspected or confirmed AHT were included. All references in the selected articles were reviewed in order to locate other studies not yet included in our sample that met the inclusion criteria of the present systematic review. The flowchart of this systematic review, following the PRISMA criteria, is shown in Fig. 1. As exclusion criteria, articles published in journals not indexed in PubMed/NIH, SciELO and LILACS/BVS databases, letters to the editor and editorials, unpublished manuscripts, dissertations, government citations or government references not published in scientific journals, and books or book chapters were not included. Regarding the designs of the studies, case reports (< 5 cases), qualitative studies, literature or systematic reviews not registered in PROSPERO, and studies without a structured methodology were excluded. Furthermore, studies that do not differentiate the pediatric from the adult population and studies conducted with cadavers or animals were not considered.

### Calibration of the research group for data collection and statistical analysis

In order to reduce the risks of selection and observation biases, the research group was calibrated during a 1-month period prior to the official collection. After resolving doubts and calibrating the research group, the official data collection was started. The selected articles were discussed in group whenever doubts emerged. Each article included in this systematic review had its level of evidence assessed according to the Oxford Centre for Evidence-Based Medicine Classification [12]. Statistical analyses were carried out using the free and open-source statistical software PSPP/GNU General Public License. Some descriptive results are presented without statistical analysis. Odds ratios (OR) were calculated to assess the association between the criteria established as predictors for suspected AHT in TBI cases, performing the pairing between victims of AHT vs. accidental TBI. Statistical significance was assessed at a p-value of < 0.05. 

## Results

A total of 462 articles were found during the first phase of the study (search in the PubMed, SciELO and LILACS databases). After application of the proposed methodology and eligibility criteria, we identified 23 articles addressing AHT in children ≤ 2 years of age in studies conducted by Latin American researchers from Argentina, Bolivia, Brazil, Colombia, Costa Rica, Cuba, Mexico, Paraguay, Peru, and Uruguay and published between 2010 and 2024. Following the established PRISMA criteria, 6 articles were selected to be included in this systematic review. The 230 bibliographic references of these 6 initially selected articles were reviewed and 28 articles written by Latin American researchers or conducted in Latin America were found among them. Of these, 10 had already been selected in the previous stage, 3 did not address AHT in children, 1 was a government communication, 6 were book chapters or non-systematic review articles, and 7 were outside the proposed study period; thus, only one article out of the 28 was added to our sample (Fig. 1). The 7 studies included in the present systematic review were conducted by Melo et al. [13], Loredo-Abdalá et al. [14], Melo et al.  [5], Díaz-Olavarrieta et al. [15], Orozco-Gómez et al. [17], Loredo-Abdalá et al. [16], and Yock-Corrales et al. [18].

The study developed by Díaz-Olavarrieta et al. [15] was prepared by Mexican researchers and had a reference hospital center in Mexico as the study site. The authors retrospectively analyzed 120 children ≤ 5 years old who were victims of TBI, identifying AHT in 11% (13) of the sample. Among the victims of AHT, the majority were girls (62%), and the median age was 8 months. The authors evaluated risk factors for suspected abuse such as pregnancy intention, regularity of prenatal care, parental age and education, alcoholism, and socioeconomic imbalances. Children who were unresponsive to stimuli (comatose) (62%) at the time of hospitalization, with reports of seizures (62%), and with retinal hemorrhages (RH) (62%) and SDH (54%) identified on cranial CT scan, were important factors for suspicion of AHT (Table 1), with high neurological disability (38%) and mortality (38%) rates.

**Table 1:** Characteristics of the studies and level of evidence of the 7 articles included in this systematic review analyzing children under 2 years of age with suspected or confirmed traumatic brain injury (TBI) due to abuse (AHT) conducted by Latin American researchers as main authors (2010–2024)

Scientific study	Study design	Sample size (AHT)	Sample size (accidental TBI)	Criteria for suspected AHT *	Level of evidence**
Díaz-Olavarrieta et al. [15]	Cross-sectional	13	107	Abnormal level of consciousness and/or seizure + RH + ICH	3
Melo et al. ) [5]	Cross-sectional	184	-	Abnormal level of consciousness and/or seizure + RH + SDH	4
Melo et al. [13]	Cross-sectional	145	50	Abnormal level of consciousness and/or seizure + RH + SDH	3
Loredo-Abdalá et al. [14]	Cross-sectional	17	-	Abnormal level of consciousness and/or seizure + RH + ICI	4
Orozco-Gómez et al. [17]	Case series	8	-	Encephalopathy (loss of consciousness and/or seizures) + RH + SDH	4
Loredo-Abdalá A et al. [16]	Cross-sectional	15	-	Abnormal level of consciousness and/or seizure + RH + ICH	4
Yock-Corrales et al. [18]	Ambispective cohort	47	345	Lowering of consciousness + convulsive seizures + SDH	3

^*^Main criteria described in the methodology or in the results of the selected articles for suspicion of AHT in cases of unwitnessed or incoherent history of trauma

^**^Oxford Centre for Evidence-Based Medicine Classification [12]

*RH*, retinal hemorrhage; *SDH*, subdural hematoma; *ICH*, intracranial hematomas; *ICI*, intracranial injuries

The studies conducted by Melo et al.  [5, 13] were international collaborations among researchers from Brazil and France. Both articles analyzed children with suspected AHT in the metropolitan region of Paris. In the first study (Melo et al. [5]), the authors found a predominance of boys (70%) with a median age of 5.8 months, and identified the criteria for indication of surgical drainage of SDH in 184 children with suspected AHT. They analyzed the Glasgow Coma Scale (GCS) score at admission, signs suggestive of intracranial hypertension (ICH), and SDH thickness on cranial CT scan. The authors found that infants with GCS ≤ 12 with signs of ICH (such as bulging fontanelles) and SDH thickness ≥ 10 mm were the main candidates for neurosurgical treatment, which included transcutaneous subdural (transfontanellar) punctures, external subdural drainage (ESD), subdural-subgaleal shunt placement (SSS), subdural-peritoneal shunt placement (SPS), craniotomies, and craniectomies. The second study carried out by Melo et al. [13] sought to identify risk factors through comparative analyses between a group of children victims of accidental TBI (*n* = 50) and another with suspected AHT (*n* = 145). The authors investigated the family history (parents’ age, family, and socioeconomic imbalances) and epidemiological criteria (address in the Paris metropolitan region) in addition to clinical criteria (clinical history, ophthalmological and neurological evaluation) as predictors of suspected abuse (Table 1). The authors identified the prevalence of boys (76%) with a median age of 5.9 months and highlighted the high rates of RH (79%), SDH (97%), and neurological disabilities (36%) in victims with suspected AHT, with a mortality rate of 4%.

Loredo-Abdalá et al. [14] retrospectively analyzed 17 children victims of AHT treated at a reference center in Mexico. The authors found a predominance of male victims (70%) with a median age of 5.7 months. The presence of seizures (82%) associated with drowsiness and loss of consciousness (65%) were clinical manifestations usually identified in these children. The authors also emphasized the high prevalence of RH (88%) and multiple brain injuries on cranial CT scan resulting from abuse (Table 1). The prevalence of neurological disabilities was 53% and the mortality rate was 35%. Another study conducted by Loredo-Abdalá et al. [16] analyzed 15 children victims of AHT, mostly males (73%) with a median age of 5 months. They highlighted risk factors in the family context and the presence of seizures (62.5%) and RH (67%) in these children (Table 1). Another study carried out in Mexico by Orozco-Gómez et al. [17] emphasized that estimating the incidence of children victims of AHT in the country is hindered by the difficulty to confirm the diagnosis, the underreporting of complaints and consequently of supporting records, and non-publication of data. The authors found a predominance of boys (5/8; 62%) with a median age of 4.1 months and highlighted three factors for suspicion of AHT: encephalopathy (drowsiness or unconsciousness), RH (mainly bilateral), and SDH (Table 1).

Yock-Corrales et al. [18] carried out a multicenter study (ambispective cohort study) with international collaboration among Latin American establishments and Asian hospital institutions, claiming to be the first study attempting to estimate the incidence of AHT in Latin America. The study evaluated 392 children with TBI, of which 12% were victims of AHT. There was a prevalence of boys (61.7%) under 2 years of age. The authors highlighted the high incidence of clinical signs such as decreased state of consciousness associated with seizures (66%) and SDH (83%) in children victims of AHT (Table 1). The mortality rate was 10.6%. Among the 7 articles included in the current systematic review, only the studies carried out by Díaz-Olavarrieta et al. [15], Melo et al. [13], and Yock-Corrales et al. [18] compared children with AHT (*n* = 205) vs. accidental TBI (*n* = 502), enabling a comparative analysis between these groups. A compilation of the results of these three studies indicated that seizures were more frequent in cases of abuse (70% vs. 46%; *p* < 0.0001) and that there was a higher prevalence of RH (78% vs. 3%; p < 0.0001) and SDH (91% vs. 29%; *p* < 0.0001) in suspected or confirmed cases of AHT (Table 2).

**Table 2:** Association (odds ratio/OR) between the criteria established as predictors for suspected abusive head trauma (AHT) recognized by Latin American researchers in cases of unwitnessed or incoherent history of trauma in children under 2 years of age (2010–2024)

Criteria for suspected AHT	Number of participants (studies included)^a^	AHT *n* (%)^b^	Accidental TBI *n* (%) ^b^	Odds ratio (95% CI)	*p*
Lowering of consciousness^c^	707 (3)	205 (95)	502 (84)	3.69 (1.8748–7.2892)	0.0002
Seizure episodes	707 (3)	205 (70)	502 (46)	2.77 (1.9573–3.9185)	< 0.0001
Retinal hemorrhages	315 (2)^d^	158 (78)	157 (3)	106.83 (40.6313–280.9057)	< 0.0001
Subdural hematoma	707 (3)	205 (91)	502 (29)	25.3 (15.0506–42.6362)	< 0.0001

*TBI*, traumatic brain injury

^a^Only studies by Díaz-Olavarrieta et al. [15], Melo et al. [13], and Yock-Corrales et al. [18] were included to calculate the odds ratio (OR) due to the possibility of comparing groups of children with AHT (*n* = 205) vs. accidental TBI (*n* = 502)

^b^Percentage of children who met the criteria assessed for suspicion of AHT

^c^Lowering of consciousness: drowsiness, lethargy or coma

^d^Although the study by Yock-Corrales et al. [18] highlighted the importance of the presence of retinal hemorrhages, it did not analyze this criterion

## Discussion

Although AHT is recognized as one of the most violent mechanisms of death and neurological disabilities in children under 2 years of age [5, 7, 13–18], many authors have highlighted a scarcity of data on the subject in low- and middle-income countries [19–22]. Research to assist in the management of children victims of TBI associated with suspicion of abuse is therefore needed [18, 23–25]. In the present systematic review, among the 7 articles that met the methodological inclusion criteria, 4 were conducted by Mexican researchers Díaz-Olavarrieta et al. [15], Loredo-Abdalá et al. [14, 16], Orozco-Gómez et al. [17], 2 resulted from a cooperation between Brazil and France Melo et al. [5, 13], and 1 article reported a study resulting from a multicenter cooperation among Latin American and Asian researchers Yock-Corrales et al. [18]. The importance of the family context in suspected abuse was analyzed in 4 articles Díaz-Olavarrieta et al. [15], Loredo-Abdalá et al. [14, 16], and Melo et al. [13], highlighting socioeconomic criteria and possible family maladjustments or imbalances as risk factors for AHT. Data regarding parental age (very young parents), unemployment and domestic violence reinforce the importance of complex and comprehensive investigations, including a detailed assessment of the entire family nucleus and of the caregivers of these children by professionals of the social service (social workers) team in cases of suspected abuse [23, 24, 26].

The low median age and low weight of children under 2 years of age facilitate for the aggressor to lift them up in the air, explaining the prevalence of AHT in this group of children. The trauma mechanism usually involves lifting the child by the armpits, compressing their thoracic region (over the ribs), followed by sudden acceleration and deceleration movements of the skull during violent shaking movements, with or without impact of the skull or other parts of the child’s body against hard surfaces [13, 27–29]. Regarding the prevalence of AHT in children victims of TBI, the studies by Díaz-Olavarrieta et al. [15] and Yock-Corrales et al. [18] showed percentages similar to previously published results, whether considering groups of children treated in reference trauma centers in Latin America (11%) [30] or in Europe (15%) [31]. In the United States of America, the incidence of AHT is estimated at 22.8 cases per 100,000 children under the age of one year [32], although it is important to note that these results may be underestimated due to the difficulty in confirming the diagnosis [6, 33–36]. Yock-Corrales et al. [18] conducted the first study attempting to estimate the incidence of AHT in Latin America, but the lack of data regarding geographic distribution made it impossible to draw inferences. Difficulties related to lack of information on geographic distribution have been reported by other Latin American researchers [11, 19–21].

An unwitnessed or incoherent history of trauma associated with altered consciousness (somnolence, lethargy or coma), seizures, RH and SDH on cranial CT scan were criteria highlighted by all Latin American authors as important factors for the suspicion of AHT in the articles included in this systematic review. These aspects are highlighted in other studies concerning the differential diagnosis between abusive and accidental TBI [23, 37–41]. In addition to a complete laboratory evaluation, the authors point that it is necessary to perform imaging tests to evaluate the entire skeleton, including CT scans of the brain. Regarding the latter imaging diagnostic method, which makes it possible to detect the presence of SDH and discuss the most appropriate treatment, only the study carried out by Melo et al. [5] explained the guidelines for the surgical management of SDH in a French reference hospital center. Determining whether a child with AHT and SDH needs neurosurgical treatment or can be managed non-surgically will depend on the experience of the pediatric neurosurgeon, the child’s GCS score at the time of admission and throughout the hospitalization period, the presence of signs suggestive of ICH, and the volume or thickness of the SDH, criteria that help in choosing among the various treatment approaches described in the medical literature [5, 42–44].

Only the studies conducted by Díaz-Olavarrieta et al. [15] and Loredo-Abdalá et al. [14] recorded the prevalence of neurological sequelae in Latin American children with AHT, which ranged from 38 to 53%. For the evaluation of this outcome (neurological disabilities), we did not include the studies by Melo et al. [5, 13] and Yock-Corrales et al. [18] because their samples were composed also of children from non-Latin American countries, the study by Loredo-Abdalá et al. [16] because it did not analyze this outcome, and the study by Orozco-Gómez et al. [17] because their objective was to evaluate ophthalmological disabilities after AHT. According to the studies by Díaz-Olavarrieta et al. [15] and Loredo-Abdalá et al. [14, 16], the only ones to report mortality exclusively in Latin American children victims of AHT, the rates varied between 20 and 38%. Considering that this is a trauma mechanism with serious consequences, as previously described, involving sudden movements of extension and flexion of the skull on immature cervical muscles and impact of the brain against rigid surfaces of the intracranial bones, AHT stands out as one of the main causes of permanent disability of neurological and/or ophthalmological nature, with high mortality rates [45, 46]. Unfortunately, considering the extensive territory of Latin America and the lack of data on these issues, it was not possible to estimate, in the current systematic review, the prevalence of neurological disabilities or the mortality rate in Latin American children victims of AHT.

Regarding the compilation of data that allowed combined analyses, only the studies by Díaz-Olavarrieta et al. [15], Melo et al. [13], and Yock-Corrales et al. [18] included in their sample children victims of TBI, comparing those with suspected or confirmed AHT to those with accidental TBI. These authors highlighted the lowering of the level of consciousness (drowsiness, lethargy or coma), seizures, RH, and SDH as factors associated with suspected AHT, criteria recognized worldwide and established as guidelines for health professionals when dealing with children victims of TBI, but suspected of abuse [23, 31, 39–41, 47–50]. According to previously established criteria for hierarchizing the levels of scientific evidence, based on the levels of evidence of the articles included in this systematic review for the identification of criteria for suspected AHT, the current study can be considered as level 3a of scientific evidence, grade of recommendation B. Systematic reviews classified as levels 2 and 3 (non-experimental) are scientific studies in which the results are observed, measured and analyzed without data manipulation. Despite the risks of possible biases due to the analysis of retrospective studies, they may be more viable to be conducted in real-world conditions, outside of laboratory or experimental environments [51, 52].

## Limitations, merits and perspectives of the study

The lack of articles published by Latin American researchers that met the methodological criteria for inclusion in this systematic review, making it impossible to understand the epidemiological characteristics such as geographic distribution, neurological disabilities, and mortality of Latin American children victims of AHT, was an important finding that prevented us from making further inferences on these topics. To reduce the risk of bias, we, the authors, followed strict criteria through calibration of the research team guided by previously established protocols, such as PROSPERO and PRISMA. To the best of our knowledge, until the date of completion of this study, this is the first systematic review study on AHT in children under 2 years of age registered in PROSPERO considering exclusively articles conducted by Latin American researchers or produced in Latin America [53]. As perspectives from the publication of this systematic review, in addition to strengthening recognized criteria to assist in the suspicion of AHT, we hope to stimulate future studies on this topic in Latin America. Considering that the criteria verified in this systematic review to establish the suspicion of AHT are recognized worldwide, and at the same time identifying the lack of reports and consequently of prosecution and punishment of the perpetrators of this crime [54], we conclude this article by quoting the researcher and writer Sven Lindqvist:—“It is not knowledge we lack. What is missing is the courage to understand what we know and to draw conclusions.” [3] We, health team professionals, need to report suspected cases of AHT, as this act is an obligation imposed by laws in several Latin American countries. It is not up to the health team to judge or punish, but rather to report. Without reporting, there will be no legal investigation and consequently there will be no one responsible for this barbaric crime, which will continue to be silenced by the connivance of many.

## Conclusion

There was a lack of scientific articles on AHT with a sufficient sample size to allow knowledge of the prevalence and geographic distribution of this event, the rates of neurological disabilities and mortality in Latin America. However, Latin American researchers emphasize and recognize that in children under 2 years of age, an unwitnessed or incoherent history of trauma associated with lowered consciousness and seizures and the presence of RH and SDH should strongly indicate suspicion of AHT. If abuse is suspected, the case should be reported to the competent legal institutions.

## Data Availability

No datasets were generated or analyzed during the current study.
